# Evolution of the *pheV*-tRNA integrated genomic island in *Escherichia coli*

**DOI:** 10.1371/journal.pgen.1011459

**Published:** 2024-10-24

**Authors:** Nguyen Thi Khanh Nhu, Brian M. Forde, Nouri L. Ben Zakour, Minh-Duy Phan, Leah W. Roberts, Scott A. Beatson, Mark A. Schembri

**Affiliations:** 1 Institute for Molecular Bioscience (IMB), The University of Queensland, Brisbane, Queensland, Australia; 2 Australian Infectious Diseases Research Centre, The University of Queensland, Brisbane, Queensland, Australia; 3 UQ Centre for Clinical Research, Faculty of Medicine, The University of Queensland, Brisbane, Queensland, Australia; 4 School of Chemistry and Molecular Biosciences, The University of Queensland, Brisbane, Queensland, Australia; Uppsala University, SWEDEN

## Abstract

*Escherichia coli* exhibit extensive genetic diversity at the genome level, particularly within their accessory genome. The tRNA integrated genomic islands (GIs), a part of the *E*. *coli* accessory genome, play an important role in pathogenicity. However, studies examining the evolution of GIs have been challenging due to their large size, considerable gene content variation and fragmented assembly in draft genomes. Here we examined the evolution of the GI integrated at *pheV*-tRNA (GI-*pheV*), with a primary focus on uropathogenic *E*. *coli* (UPEC) and the globally disseminated multidrug resistant ST131 clone. We show the gene content of GI-*pheV* is highly diverse and arranged in a modular configuration, with the P4 integrase encoding gene *intP4* the only conserved gene. Despite this diversity, the GI-*pheV* gene content displayed conserved features among strains from the same pathotype. In ST131, GI-*pheV* corresponding to the reference strain EC958 (EC958_GI-*pheV*) was found in ~90% of strains. Phylogenetic analyses suggested that GI-*pheV* in ST131 has evolved together with the core genome, with the loss/gain of specific modules (or the entire GI) linked to strain specific events. Overall, we show GI-*pheV* exhibits a dynamic evolutionary pathway, in which modules and genes have evolved through multiple events including insertions, deletions and recombination.

## Author summary

Genomic islands (GIs) are key contributors to *E*. *coli* pathogenesis. However, studies on the evolution of GIs are limited, mostly due to their large size, variation, and fragmented assembly in draft genomes. Here we investigated the evolution of the GI-*pheV* genomic island. We showed that the presence of GI-*pheV* is primarily associated with pathogenic *E*. *coli*, with the greatest diversity in gene content observed among strains from different pathotypes. The GI-*pheV* in UPEC contains uropathogenicity-associated virulence and fitness encoding modules, which have either co-evolved with the island or evolved independently, possibly due to different selective pressures. Focused investigation of GI-*pheV* in ST131, the most globally dominant antibiotic resistant UPEC clone, revealed loss or gain of genes and modules via recombination during its evolution.

## Introduction

*Escherichia coli* is an extremely diverse species. Some strains can colonize the gastrointestinal tract as harmless commensals, while others possess distinct sets of virulence genes that enable them to cause disease [1,2]. *E*. *coli* is classified based on virulence and site of infection, ranging from commensal strains to intestinal pathogenic *E*. *coli* (InPEC) and extra-intestinal pathogenic *E*. *coli* (ExPEC). InPEC and ExPEC can be further grouped into a range of pathotypes: InPEC—enteropathogenic *E*. *coli* (EPEC), enterohemorrhagic *E*. *coli* (EHEC), enterotoxigenic *E*. *coli* (ETEC), enteroaggregative *E*. *coli* (EAEC), enteroinvasive *E*. *coli* (EIEC), adherent-invasive *E*. *coli* (AIEC) and enteroaggregative and hemorrhagic *E*. *coli* (EAHEC); ExPEC—uropathogenic *E*. *coli* (UPEC), neonatal meningitis *E*. *coli* (NMEC) and avian pathogenic *E*. *coli* (APEC) [1,2].

The *E*. *coli* genome exhibits extensive plasticity, with ~55% gene conservation observed across strains from different pathotypes [3,4]. Moreover, pathogenic *E*. *coli* strains have a more diverse gene content than their commensal counterparts [3,5]. Even in a single multidrug resistant ExPEC lineage such as the globally disseminated ST131 clone, the core genome accounts for only ~75% of the average genome size [6,7]. Studies on the evolution of ST131 showed that the lineage is comprised of three distinct clades, namely clade A, clade B and clade C. Strains from Clade C form a globally dominant fluoroquinolone resistant group, and can be further divided into two subclades (C1 and C2), of which strains from C2 are associated with the presence of the extended spectrum beta-lactamase CTX-M-15 gene [6–12].

The accessory genome, defined as genomic elements present in only a subset of *E*. *coli* strains, includes prophages, plasmids, integrative and conjugative elements (ICEs), and genomic islands (GIs); these elements play an important role in *E*. *coli* evolution, adaptation, resistance and virulence [13–21]. The most striking difference between genomes of non-pathogenic and pathogenic *E*. *coli* lies in GIs, which are often >100 kb in size and generally contain multiple virulence and/or fitness genes acquired through lateral gene transfer [3,5,14,22,23]. GIs possess various combinations of the following features: (i) a G+C content different from the average G+C content of the core genome, (ii) a modular arrangement of smaller mobile elements, (iii) differing levels of stability, (iv) a strong association with pathogenic strains, (v) integration sites near tRNA genes, and (vi) multiple virulence genes (also referred to as pathogenicity islands—PAIs) or resistance genes (associated with resistance islands) [16,24–28]. In *E*. *coli*, hotspots for the integration of GIs are usually located at the *selC*-tRNA, *leuX*-tRNA, *thrW*-tRNA, *serT*-tRNA, *serW-*tRNA, *asnT*-tRNA, *pheV*-tRNA and *pheU*-tRNA [23]. Among these sites, *pheV-*tRNA represents the major integration hotspot for GIs containing virulence factors associated with pathogenic *E*. *coli* such as UPEC, EHEC, EPEC and ETEC [23,29–31]. For example, the GI-*pheV* found in CFT073 (denoted CFT073_GI-*pheV*) is found more frequently in strains associated with acute pyelonephritis and cystitis than in fecal strains [29]. In addition, GIs containing the locus of enterocyte effacement (LEE), which encodes a type III secretion system, are inserted at *pheV*-tRNA in many EHEC and EPEC strains [18,32]. Finally, *pheV*-tRNA (and *pheU*-tRNA) are also insertion hotspots for ICEs such as ICE*Ec2*, which can comprise both virulence and antibiotic resistance genes [33].

Despite the wealth of knowledge on *E*. *coli* genomics, the study of GIs and their evolution has been challenging due to their large size, diverse gene content, highly mosaic structure and fragmented assembly in draft genomes [34]. Here, we examined the GI integrated at *pheV-*tRNA (GI-*pheV*) to understand its contribution to *E*. *coli* evolution and virulence, with a focus on its evolution in the ST131 lineage.

## Results

### The presence of GI-*pheV* is associated with pathogenic *E*. *coli*

**Fig 1:**
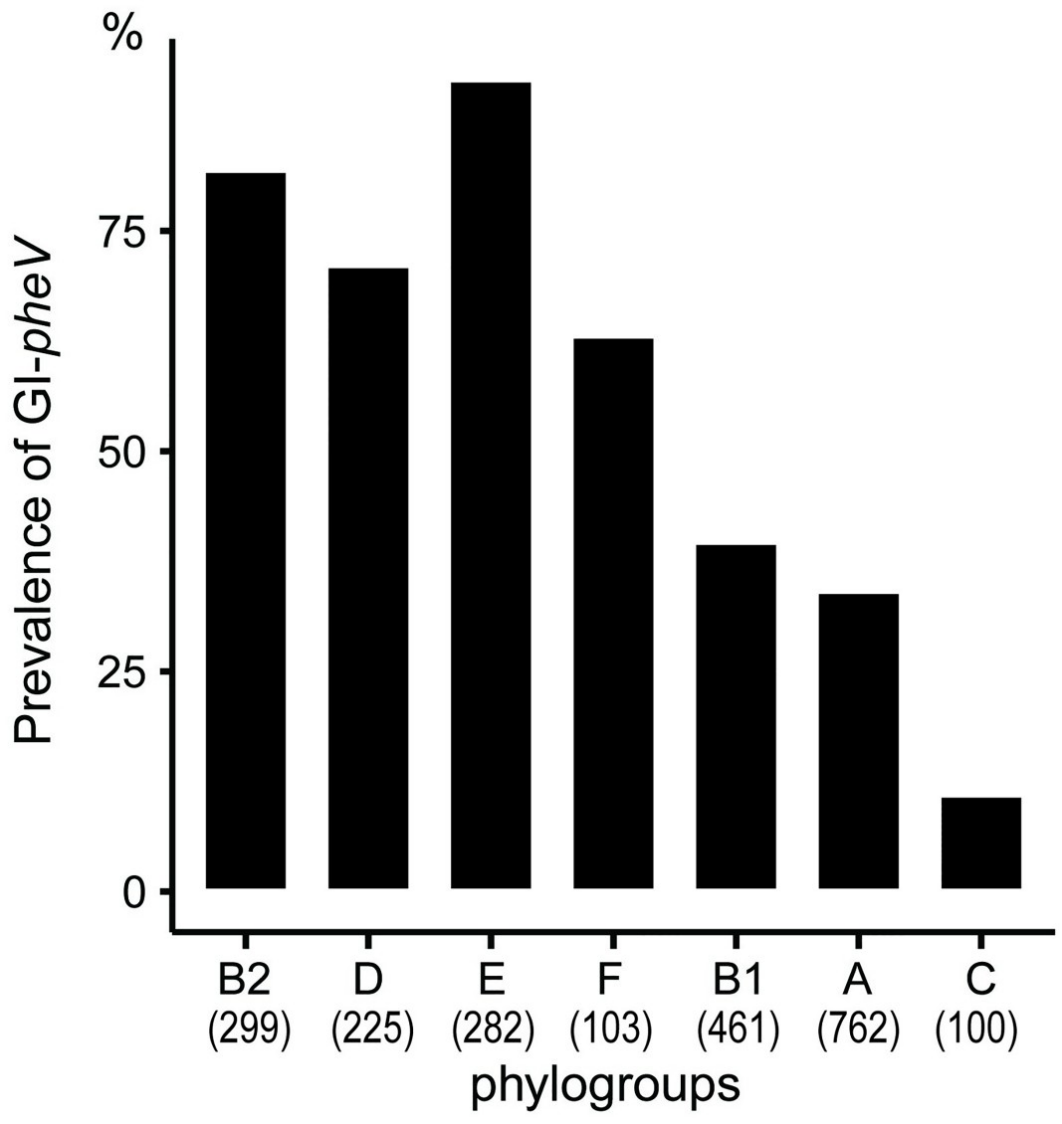
Prevalence of GI-*pheV* in the 2,382 *E*. *coli* complete genomes from the RefSeq Database. The total number of *E*. *coli* genomes in each phylogroup is shown in brackets.

We began by determining the prevalence of GI-*pheV* in 2,328 *E*. *coli* complete genomes from NCBI Reference Sequence Database (RefSeq, retrieved on 23/12/2022) based on the presence of *pheV*-tRNA, the integrase encoding gene *intP4* and a coding sequence located upstream of the *pheV*-tRNA. Based on these criteria, GI-*pheV* was identified in 52% (1,242/2,383) *E*. *coli* complete genomes (S1 Table). However, the prevalence of GI-*pheV* was different among *E*. *coli* phylogroups and sequence types (STs; Figs 1 and S1). GI-*pheV* was most prevalent in phylogroups associated with pathogenesis including ExPEC-associated phylogroups B2 (82%), D (71%) and F (63%), and EHEC-associated phylogroup E (92%). Furthermore, GI-*pheV* was present in >90% of strains from major ExPEC STs such as ST131, ST73 and ST127, and Shiga toxin-producing O157:H7 *E*. *coli* ST11 (S1 Fig). In contrast, GI-*pheV* was present in <40% of non-pathogenic phylogroups A (34%) and B1 (39%).

Next, we established a reference set of 66 completely sequenced *E*. *coli* genomes representing a range of phylogroups and STs available on the NCBI Genbank database, and comprising non-pathogenic (n = 23) and pathogenic (n = 35) strains (S2 Table). In total, GI-*pheV* was detected in 41/66 (62%) genomes from this dataset. The location, size and direct repeat sequences for the 41 identified GI-*pheV* are listed in Table 1. GI-*pheV* was found in all pathogenic strains, but only in 26% (6/23) of non-pathogenic strains, indicating a strong association of GI-*pheV* and pathogenic *E*. *coli* (*P* < 0.0001, Chi-square test; S2 Table). GI-*pheV* was present in all phylogroup E (n = 12), phylogroup D (n = 2) and phylogroup F (n = 3) strains, as well as 8/15 (53%) phylogroup B1 strains and 12/19 (63%) B2 strains. In contrast, GI-*pheV* was only identified in 4/15 (27%) phylogroup A strains, which comprise non-pathogenic strains. The size of the 41 GI-*pheV* was extremely variable, ranging from 9.7 kb (11368_GI-*pheV*) to 110 kb (55989_GI-*pheV*) (Table 1). Among the 41 identified GI-*pheV*, only 27 (66%) contained direct repeats flanking the GI (Table 1). These sequences upstream (*attL*) and downstream (*attR*) of the GI-*pheV* sequences were mostly either a 23-bp direct repeat (16 strains) or a 19-bp direct repeat (10 strains), and include part of the *pheV-*tRNA gene sequence (Table 1). GI-*pheV* that lacked a recognisable *attR* (mean 23.5 kb, Q1 –Q3 18 kb– 32.1 kb) were significantly smaller than those where an *attR* (mean 75 kb, Q1 –Q3 52.1 kb– 87.4 kb) was identified (*P* < 0.0001, unpaired *t-*test).

**Table 1:** List of GI-*pheV* from *E*. *coli* complete genomes.

Strain	Serotype	Phylo group	MLST	Pathotype ^1^	Host	Source	Disease	GI-*pheV* size	GI start ^2^	GI end ^2^	*attR* ^3^
APEC O1	O1:K1:H7	B2	95	APEC	turkey	lung	colisepticemia	55,888	3,305,017	3,360,904	TTCGATTCCGAGTCCGG-CACCA
IAI39	O7	D	62	UPEC	human	urine	pyelonephritis	38,493	3,545,466	3,583,958	no direct repeat
S88	O45:H7	B2	95	NMEC	human	CSF	meningitis	55,896	3,219,801	3,275,696	TTCGATTCCGAGTCCGG-CACCA
ABU 83972	Ont:K5 ^a^	B2	73	non-path	human	urine	ABU	74,987	3,326,741	3,401,727	TT**TC**ATTCCGA**T**TCCGGGCACCA
CE10	O7:K1	F	62	NMEC	human	CSF	meningitis	46,016	3,487,188	3,533,203	no direct repeat
CFT073	O6:H1	B2	73	UPEC	human	blood	pyelonephritis	104,676	3,406,225	3,510,900	TT**TC**ATTCCGA**T**TCCGGGCACCA
D i14	O6	B2	73	UPEC	dog	stool	-	74,987	3,293,346	3,368,332	TT**TC**ATTCCGA**T**TCCGGGCACCA
D i2	O6	B2	73	UPEC	human	stool	-	74,987	3,293,346	3,368,332	TT**TC**ATTCCGA**T**TCCGGGCACCA
EC958	O25b:H4	B2	131	UPEC	human	urine	urosepsis	75,083	3,284,808	3,359,890	TT**TC**ATTCCGA**T**TCCGGGCA**T**CA
JJ1886	-	B2	131	UPEC	human	urine	urosepsis	73,646	3,336,265	3,409,910	TT**TC**ATTCCGA**T**TCCGGGCACCA
Nissle 1917	O6:K5:H1	B2	73	non-path	human	-	-	76,239	1,729,621	1,805,859	TT**TC**ATTCCGA**T**TCCGGGCACCA
UMN026	O17:H18	D	597	UPEC	human	urine	cystitis	82,784	3,445,924	3,528,707	TT**TC**ATTCCGA**T**TCCGGGCACCA
ATCC 8739	O146	A	1120	non-path	-	-	-	19,321	783,046	802,366	no direct repeat
ED1a	O81	B2	452	non-path	human	stool	commensal	52,124	3,374,807	3,426,930	TTCGATTCCGAGTCCGG-CACCA
H10407	O78:H11	A	48	ETEC	human	stool	diarrhea	73,050	3,413,160	3,486,209	TTCGATTCCGAGTCCGG-CACCA
RM12581	O145:H28	E	-	EHEC	-	food	-	13,862	3,750,629	3,764,490	no direct repeat
RM12761	O145:H28	E	-	EHEC	-	food	-	32,114	3,665,863	3,697,976	no direct repeat
RM13514	O145:H28	E	-	EHEC	human	stool	-	13,862	3,750,632	3,764,493	no direct repeat
RM13516	O145:H28	E	-	EHEC	human	stool	-	32,114	3,665,859	3,697,972	no direct repeat
12009	O103:H2	B1	17	EHEC	human	stool	diarrhea	89,251	3,627,082	3,716,332	TTCGATTCCGAGTCCGG-CACCA
CB9615	O55:H7	E	335	EPEC	human	stool	diarrhea	49,355	3,675,873	3,725,227	TTCGATTCCGAGTCCGG-CACCA
E2348/69	O127:H6	B2	15	EPEC	human	stool	diarrhea	29,418	3,338,615	3,368,032	**AAAGTTCT**CGAGTCCGGGCACCA
EC4115	O157:H7	E	11	EHEC	human	stool	diarrhea	22,215	3,956,357	3,978,571	no direct repeat
EDL933	O157:H7	E	-	EHEC	-	food	-	23,531	3,919,272	3,942,802	no direct repeat
RM12579	O55:H17	E	335	EPEC	human	urine	-	43,775	3,589,936	3,633,710	TTCGATTCCGAGTCCGG-CACCA
Sakai	O157:H7	E	11	EHEC	human	stool	diarrhea	23,530	3,851,960	3,875,489	no direct repeat
SS17	O157:H7	E		STEC	cow	rectal	-	22,215	3,908,165	3,930,379	no direct repeat
TW14359	O157:H7	E	11	EHEC	human	stool	diarrhea	23,530	3,911,083	3,934,612	no direct repeat
Xuzhou21	O157:H7	E	96	EHEC	human	stool	diarrhea	23,530	3,779,319	3,802,848	no direct repeat
55989	O104:H4	B1	678	EHEC	human	stool	diarrhea	110,510	3,339,841	3,450,350	TTCGATTCCGAGTCCGG-CACCA
2009EL-2050	O104:H4	B1	678	EHEC	human	stool	diarrhea	86,738	947,836	861,099	TTCGATTCCGAGTCCGG-CACCA
2009EL-2071	O104:H4	B1	678	EHEC	human	stool	diarrhea	87,388	937,496	850,109	TTCGATTCCGAGTCCGG-CACCA
2011C-3493	O104:H4	B1	678	EHEC	human	stool	diarrhea	87,802	944,386	856,585	TTCGATTCCGAGTCCGG-CACCA
E24377A	O139:H28	B1	1132	EPEC	human	stool	diarrhea	101,767	3,325,510	3,427,276	TT**TT**ATTCCGAGTCCGGGCACCA
UMNK88	O149	A	100	ETEC	porcine	stool	-	38,928	3,543,887	3,582,814	TTCGATTCCGAGTCCGG-CACCA
42	O44:H18	D	414	EAEC	human	stool	diarrhea	54,575	3,387,125	3,441,699	TTCGATTCCGAGTCCGG-CACCA
536	O6:H31	B2	127	UPEC	human	urine	pyelonephritis	48,805	3,128,084	3,176,888	TTCGATTCCGAGTCCGG-CACCA
11128	O111-H-	B1	16	EHEC	human	stool	diarrhea	54,250	3,705,241	3,759,490	TTCGATTCCGAGTCCGG-CACCA
11368	O26:H11	B1	21	EHEC	human	stool	diarrhea	9,995	4,029,587	4,039,581	no direct repeat
REL606		A	93	non-path	human	stool	-	15,141	2,996,021	3,011,161	TTCGATTCCGAGTCCGG-CACCA
SMS-3-5	O19:H34	F	354	non-path	-	water	-	91,940	3,197,159	3,289,098	TT**TT**ATTCCGAGTCCGGGCACCA

^1^ Abbreviations: ABU, asymptomatic bacteriuria; AIEC, adherent-invasive *E*. *coli*; APEC, avian pathogenic *E*. *coli*; EAEC, enteroaggregative *E*. *coli*; EHEC, enterohemorrhagic *E*. *coli*; EPEC, enteropathogenic *E*. *coli*; ETEC, enterotoxigenic *E*. *coli*; NMEC, neonatal meningitis *E*. *coli*; UPEC, uropathogenic *E*. *coli*; non-path, non-pathogenic.

^2^ GI start was identified as the first 5’ base of *pheV*-tRNA, GI end was either the end of *attR* or the approximation of the last nucleotide before the conserved region compared to K-12 backbone.

^3^ Nucleotides different from the last 23 bp of tRNA-*pheV* (*attL*) was highlighted as bold; nucleotide deletion is the dash.

^a^ nt: non-typeable

### GI-*pheV* can be classified by the type of *intP4* integrase gene

Core-genome analysis of the 41 GI-*pheV* sequences from our dataset revealed that the only conserved coding sequence in all completely sequenced GI-*pheV* was the *intP4* gene (S2 Fig). The *intP4* gene encodes a P4 integrase type tyrosine site-specific recombinase and is located immediately adjacent to the *pheV*-tRNA. Therefore, to examine the evolution of GI-*pheV*, we analyzed the phylogenetic relationship of the *intP4* gene from the 41 GI-*pheV* described above. The most divergent *intP4* genes were identified in GI-*pheV* from the environmental *E*. *coli* strain SMS-3-5 and the commensal *E*. *coli* strain REL606, sharing approximately 46.3% and 85% nucleotide conservation with *intP4* in other GI-*pheV*, respectively. In contrast, the 39 *intP4* genes from the remaining GI-*pheV* were very closely related (95% of *intP4* with ≥ 94% nucleotide sequence conservation). Of note, we observed a similarly high sequence conservation of *intP4* among the 1,242 GI-*pheV*-positive *E*. *coli* complete genomes in the larger RefSeq database; 98% of *intP*4 genes shared > 94% nucleotide sequence conservation (S3 Fig), indicating that the analysis of 39 *intP4* genes was sufficient to capture major hallmarks of GI-*pheV* variation in *E*. *coli*.

**Fig 2:**
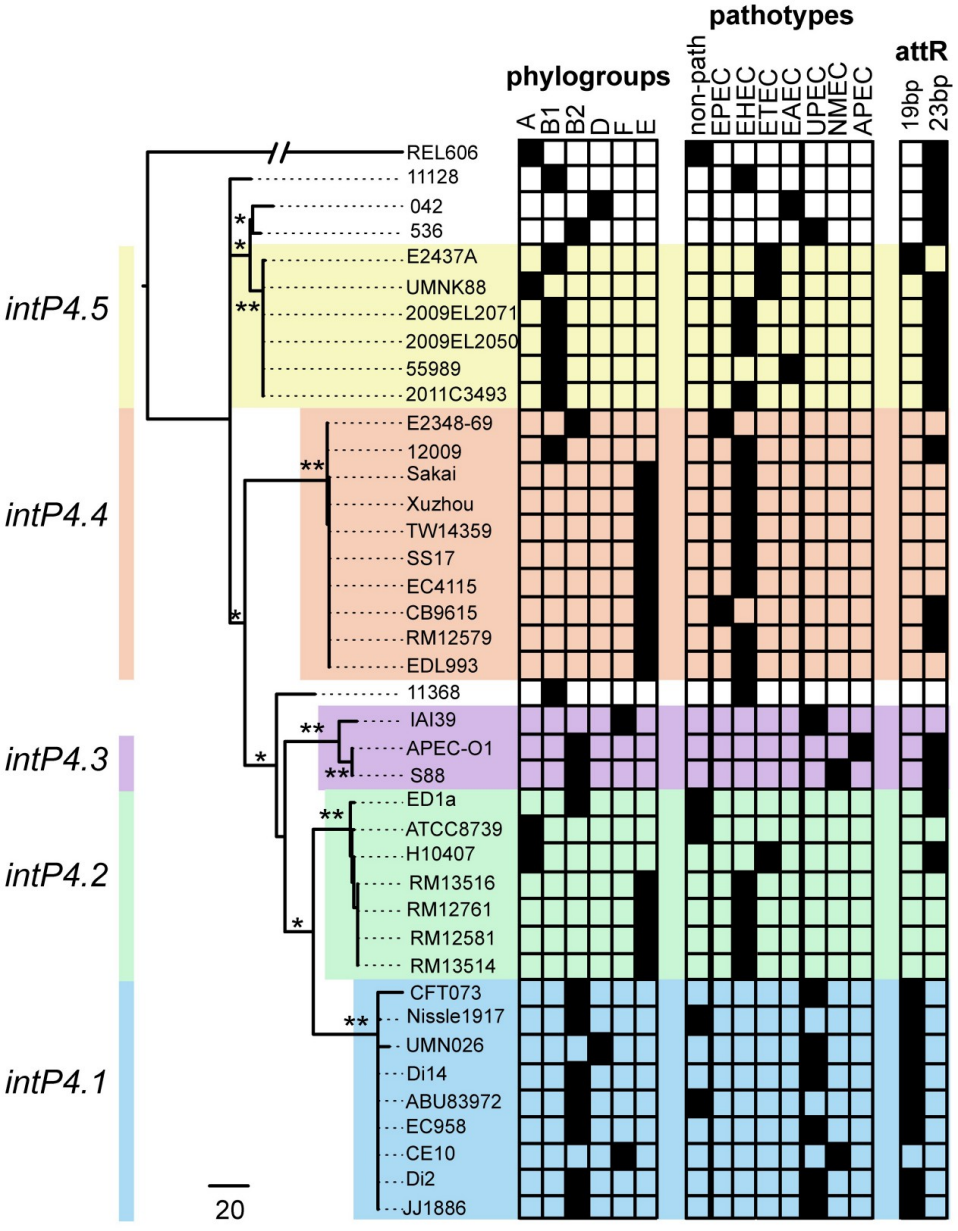
Phylogeny of *intP4* in the 40 GI-*pheV* from the reference set of 66 *E*. *coli* complete genomes. A phylogenetic tree of 40 *intP4* nucleotide sequences (except SMS-3-5_*intP4*) was constructed using RaxML, with 1,000 bootstraps, and the scale as the number of SNPs. The *intP4* gene clustered into 5 main alleles, namely *intP4*.*1* –*intP4*.*5* (coloured across each group for clarity). Highly supported branches are marked with one (*) or two (**) asterisks based on its bootstrapped value (>50% and >90%, respectively). *E*. *coli* phylogroups, pathotypes and the 3’ end direct repeat (*attR*) of GI-*pheV* are shown on the right of the *intP4* phylogeny (indicated by black boxes). Strain types: non-path, non-pathogenic; EPEC, Enteropathogenic *E*. *coli*; EHEC, Enterohemorrhagic *E*. *coli*; ETEC, Enterotoxigenic *E*. *coli*; EAEC, Enteroaggregative *E*. *coli*; UPEC, Uropathogenic *E*. *coli*; NMEC, neonatal meningitis associated *E*. *coli*; APEC, Avian pathogenic *E*. *coli*.

Phylogenetic analysis revealed that most of 39 *intP4* genes of GI-*pheV* could be clustered into 5 main alleles, that we have named *intP4*.*1* to *intP4*.*5* (Fig 2). The nucleotide sequence of each *intP4* allele was highly similar (< 1% divergence), but divergence between *intP4* alleles ranged from 4–6%. The *intP4* alleles displayed a phylogroup association; most alleles in phylogroup B1 strains belonged to *intP4*.*5*, most alleles in phylogroup B2 strains belonged to *intP4*.*1* or *intP4*.*3*, and most alleles in phylogroup E strains belonged to *intP4*.*2* or *intP4*.*4*. The *intP4* alleles were also largely congruent with strain pathotypes; e.g. *intP4* from InPEC strains comprised *intP4*.*2*, *intP4*.*4* and *intP4*.*5* alleles, while *intP4* from ExPEC strains comprised *intP4*.*1* and *intP4*.*3* alleles (Fig 2).

### GI-*pheV* gene content is associated with *intP4* integrase type

**Fig 3:**
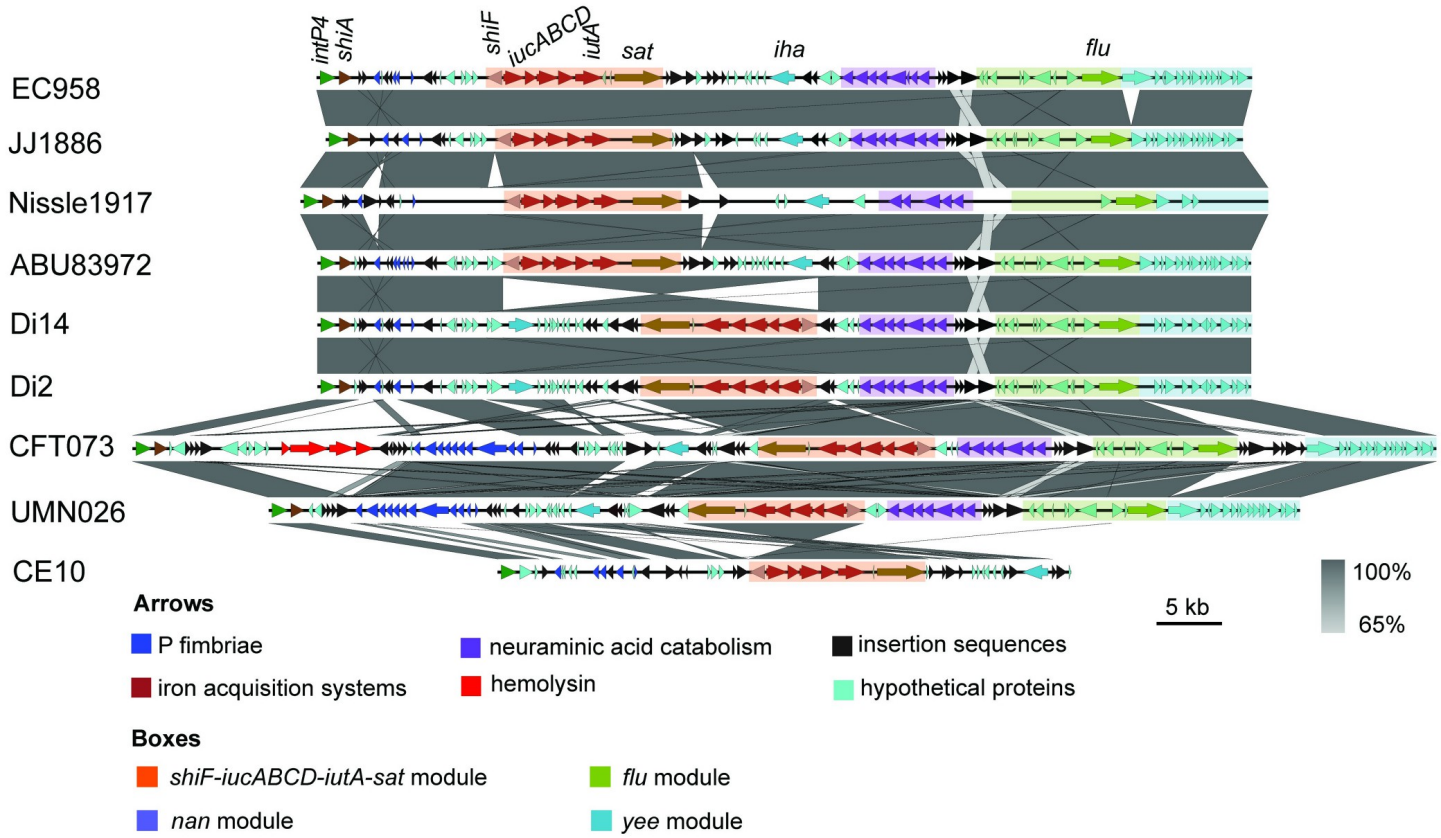
Gene content of intP4.1_GI-*pheV*. Gene content of intP4.1_GI-*pheV* visualized based on an alignment of the entire GI using Easyfig (drawn to scale). The black to grey gradient between the GI indicates their pairwise nucleotide conservation. Conserved genes and gene modules are colour coded as indicated in the text. Insertion sequences (transposons, transposases and other mobile elements) are drawn as black arrows.

Given the role of the P4 integrase in GI-*pheV* mobilization, we hypothesized that GI-*pheV* containing the same *intP4* allele would have the same or similar gene content. In fact, GI-*pheV* harbouring different *intP4* groups have different gene content (S4 Fig). Comparative analyses of *intP4*.*1*-harbouring GI-*pheV* (hereafter referred as intP4.1*_*GI*-pheV*) revealed that they possessed very similar gene content, irrespective of the orientation and location of the genes in the GI (Fig 2). Similar to other GIs, intP4.1_GI-*pheV* has a mosaic structure comprising several gene modules, which we define as clustered coding sequences within a GI separated by insertion sequences (Fig 3). The intP4.1_GI-*pheV* contained genes encoding various fitness factors and/or virulence factors associated with uropathogenesis, including a complete or partial pyelonephritis-associated P fimbriae gene cluster (*pap*), the secreted autotransporter serine protease gene (*sat*), an adhesin gene (*iha*), the aggregation- and biofilm-associated antigen 43 gene (*flu*), the aerobactin siderophore biosynthesis gene cluster (*iucABCD*) and the aerobactin receptor (*iutA*) (Fig 3). GI-*pheV* from six out of the eight UPEC genomes examined that contained *intP4* belonged to *intP4*.*1*. While a full *pap* operon is present in CFT073_GI-*pheV* and UMN026_GI-*pheV*, other GI-*pheV* in the UPEC strains examined, including EC958_GI-*pheV* and JJ1886_GI-*pheV*, only harboured remnants of the *pap* locus. All intP4.1_GI-*pheV* except CE10_GI-*pheV* contained a 19-bp imperfect direct repeat (with a nucleotide substitution) at the 3’ of the island (Table 1). CE10_GI-*pheV* was the smallest intP4.1_GI-*pheV* examined (46 kb) and contained the fewest virulence-associated genes (Fig 3).

The intP4.2_GI-*pheV* was identified in strains belonging to three different phylogenetic groups: A, B2 and E (Fig 1). The core component of intP4.2_GI-*pheV* was restricted to the *fec* cluster (which encodes a ferric citrate transport system). The remaining gene content of intP4.2_GI-*pheV* was variable, with a remnant of the *yee* module (which contains coding sequences related to the CP4-44 prophage) present in H10407_GI-*pheV* and ED1a_GI-*pheV*, and a remnant of the *flu* module present in H10407_GI-*pheV* (S4 and S5 Figs).

The intP4.3_GI-*pheV* contains a number of genes including *tia* (encoding an invasion determinant of ETEC), a full *pap* operon with the same gene composition as the one in CFT073_GI-*pheV* but with extensive sequence variation in the *papA* gene encoding the major pilin protein PapA (67% amino acid sequence identity) and the *papX* gene encoding the PapX regulator (22% amino acid sequence identity), *pgt* (encoding a phosphoglycerate transport protein), *ireA* (encoding an iron-responsive element), *ibr* (encoding an immunoglobulin-binding protein) and a remnant of the *yee* module (S4 and S5 Figs). The *ibr* and *yee* modules were also identified in intP4.5_GI-*pheV* (S4 Fig). A typical intP4.5_GI-*pheV*, for example 55989_GI-*pheV*, contained a gene encoding a putative DNA helicase, a gene encoding a serine protease (*pic*), and a gene cluster encoding a putative type VI secretion system (T6SS) (S4 and S5 Figs).

The gene content of intP4.4_GI-*pheV* was highly conserved in the strains examined. Strains that possessed intP4.4_GI-*pheV* belonged to phylogroup E and were either EPEC or EHEC. The intP4.4_GI-*pheV* shared the same gene content as the previously described Sakai_GI-*pheV* (SpLE3) (also known as OI-122 in EDL933) [35–37], which contains EHEC-associated virulence factors including an adherence/lymphocyte inhibition factor (*efa1/lifA*), an enterotoxin homologous to the *Shigella flexneri* SenA (*ent*) and a virulence factor associated with *Salmonella typhimurium* survival in macrophages (*pagC*) (S4 and S5 Figs). Most intP4.4_GI-*pheV* did not contain direct repeat (*attR*) sequences, the exceptions being 12009_GI-*pheV*, CB9615_GI-*pheV* and RM12579_GI-*pheV*, which possessed a 23-bp imperfect *attR* (Fig 1). These GI-*pheV* were larger than the other intP4.4_GI-*pheV*, and contained remnants of the *flu* and *yee* modules in addition to components of Sakai_GI-*pheV*. In strain 12009, the core LEE (encoding T3SS effectors and structure proteins—*esp*, *sep*, *ces*, and the translocated intimin receptor—*tir*) is located downstream of the 12009_GI-*pheV attR* direct repeat, suggesting its independent acquisition in this strain.

### Gene modules and individual genes of intP4.1_GI-*pheV* have evolved differently

**Fig 4:**
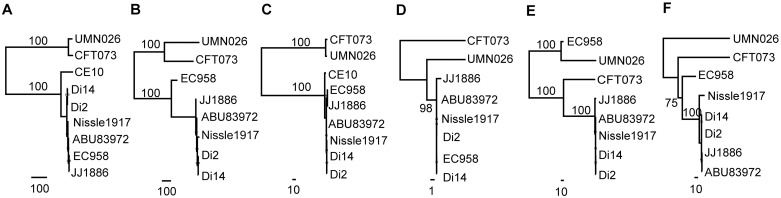
Phylogenetic relationship of intP4.1_GI-*pheV* and its modules. Whole GI-*pheV* or gene modules were aligned using Mauve or ClustalO, respectively. Phylogenetic trees were constructed using RAxML, with 1,000 bootstraps. Highly supported branches are shown with bootstrapped values and scales indicating the number of SNPs. Phylogenetic trees depicted as follows: (A) nine intP4.1_GI-*pheV* (phylogenetic tree constructed based on a 30,859 bp—core GI-*pheV*). (B) eight intP4.1_GI-*pheV* (phylogenetic tree excluding CE10_GI-*pheV*, constructed based on a 64,922-bp—core GI-*pheV*). (C) *shiF_iucABCD_iutA_sat* module. (D) *nan* module. (E) *flu* module. (F) *yee* module.

The analyses described above demonstrate that intP4.1_GI-*pheV* is largely restricted to UPEC. Indeed, analysis of the 1,242 GI-*pheV*-positive *E*. *coli* complete genomes in the RefSeq database revealed the *intP4*.*1* allele was predominantly found in UPEC/ExPEC associated phylogroups B2 (57%), D (49%) and F (42%) compared to phylogroup A (6%) (S1 Table). Thus, the remainder of this study focused on this GI to understand its evolution and contribution to UPEC virulence. Phylogenetic analysis of nine intP4.1_GI-*pheV* nucleotide sequences showed that intP4.1_GI-*pheV* could be divided into two well-supported clusters, of which cluster 1 comprised CFT073_GI-*pheV* and UMN026_GI-*pheV* and cluster 2 comprised the remaining seven GI-*pheV* (Fig 4A). The integrase gene of CFT073_GI-*pheV* and UMN026_GI-*pheV* were the most divergent within the *intP4*.*1* group, differing from other strains by 13 SNPs and 6 SNPs, respectively. Given the comparative small size of CE10_GI-*pheV*, we removed it from our dataset and repeated the analysis. The resulting investigation based on a 64,922-bp core GI-*pheV* produced a similar overall phylogeny, with the exception that EC958_GI-*pheV* segregated separately from the other GIs (Fig 4B).

The intP4.1_GI-*pheV* possesses a mosaic structure with several modules separated by insertion sequences (Fig 3). To examine the coevolution of these elements, we analyzed the phylogenetic relationship between each module, designated as: (i) the *shiF_iucABCD_iutA_sat* module (14,133 bp), (ii) the *nan* module (8,905 bp), (iii) the *flu* module (11,415 bp), and (iv) the *yee* module (8,951 bp) (Fig 3). Overall, the modules from CFT073_GI-*pheV* and UMN026_GI-*pheV* clustered together with the exception of the *flu* module, where UMN026_*flu* possessed a closer relationship to EC958_*flu* (Fig 4). The *nan* module showed the highest conservation (99.7% nucleotide sequence conservation), while other modules are more diverse, with nucleotide sequence identity ranging from 95%–97% (Fig 4).

**Fig 5:**
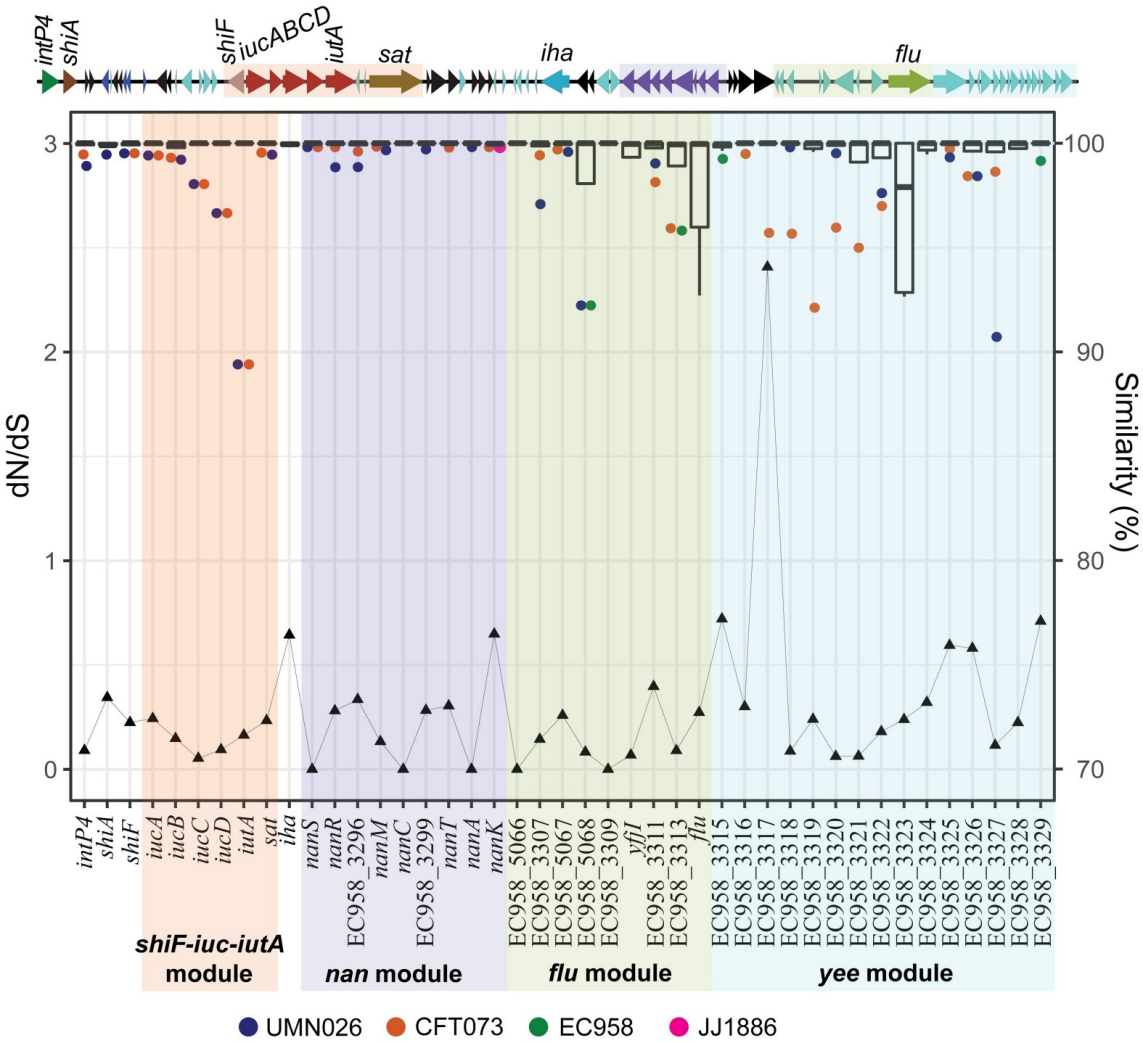
Nucleotide conservation and selection pressures within intP4.1_GI-*pheV*. The average dN/dS ratio is indicated with a triangle (value range on the left y-axis). Nucleotide similarity, compared to the consensus sequences, is shown with box plots (value range on the right y-axis), with the middle line showing the group median and the coloured dots as the group outliers (defined as any value outside 1.5 times the interquartile range below the first quartile).

Next, we investigated the genetic variation between individual genes within intP4.1_GI-*pheV*. Nucleotide sequence conservation varied among the individual genes examined, despite their preserved modular location (Fig 5). The major contributor to nucleotide diversity was the iron-acquisition genes from CFT073 and UMN026, suggesting they have evolved under strain-specific selective pressures (Fig 5). In the *shiF-iucABCD-iutA-sat* module, although *shiF*, *iucA*, *iucB* and *sat* share more than 99.5% nucleotide sequence identity, *iucC*, *iucD* and *iutA* from CFT073_GI-*pheV* and UMN026_GI-*pheV* are comparatively diverse (98%, 96% and 89% nucleotide sequence identity compared to their corresponding genes in other intP4.1_GI-*pheV*, respectively) (Fig 5). Similarly, the SNP frequencies of individual genes (determined by the number of SNPs over the entire gene length) varied considerably across GI-*pheV* (S3 Table). While *shiF*, *iucA*, *iucB* and *sat* had 5.8–7.4 SNPs/Kb, the SNP frequencies were approximately 5 and 17 times higher in *iucD* and *iutA*, respectively. Although previous reports have found diversifying (positive) selection in ferrisiderophore receptors from other organisms such as *Pseudomonas aeruginosa* [38], pairwise dN/dS analysis between CFT073 and EC958 suggests that most genes in intP4.1_GI-*pheV* are under negative (purifying) selection (dN/dS < 1) (Fig 5). The most likely explanation for higher SNP frequencies in *iucD* and *iutA* is by recombination of a region overlapping both *iucD* and *iutA* prior to acquisition of the *shiF-iucABCD-iutA-sat* module by the last common ancestor of CFT073_GI-*pheV* and UMN026_GI-*pheV*. Overall, this analysis supports the hypothesis that individual modules of intP4.1_GI-*pheV* have evolved independently via mutation and/or recombination.

### A specific GI-*pheV* is common to ST131

**Fig 6:**
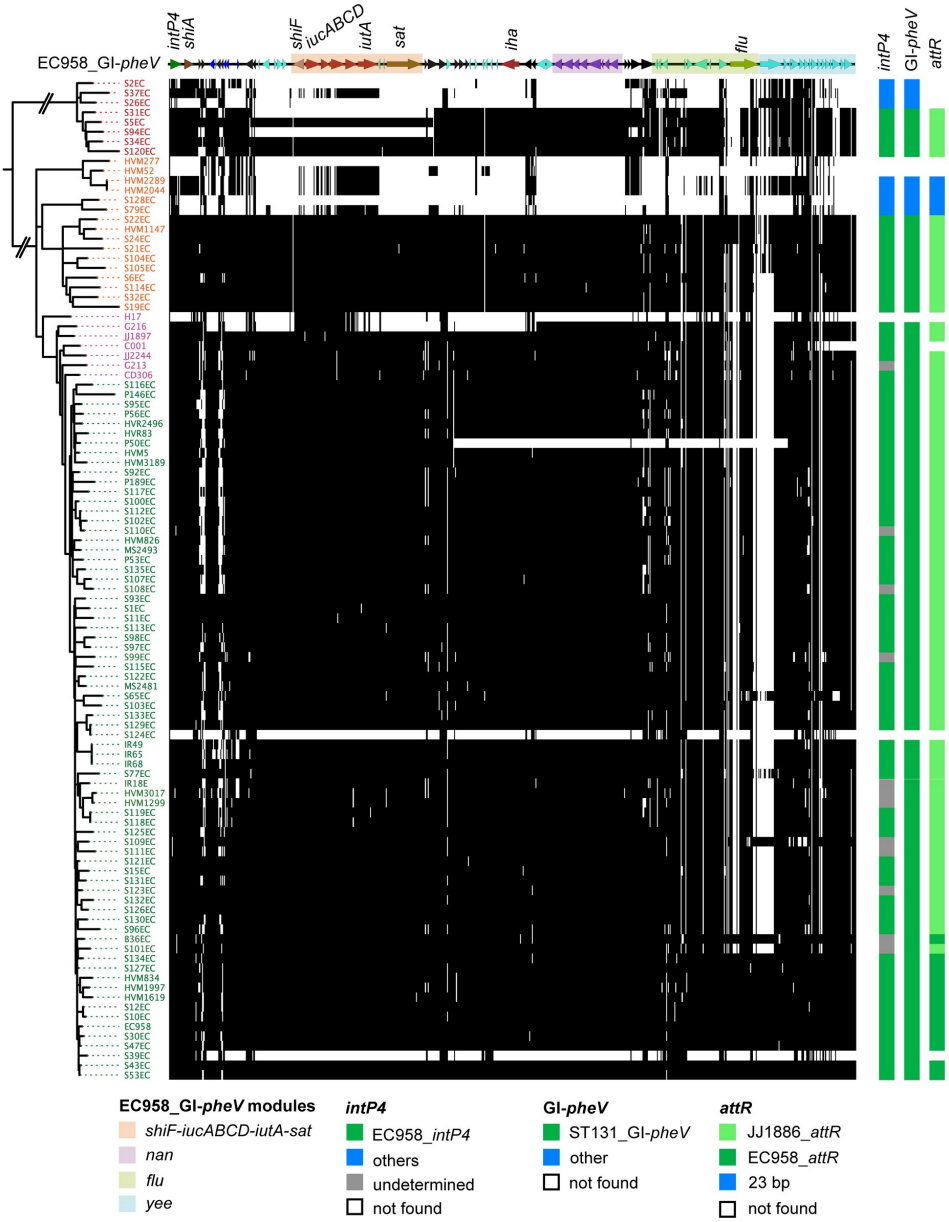
The prevalence of ST131_GI-*pheV* in ST131. The gene content of EC958_GI-*pheV* is shown along the x-axis, with the annotation on top (drawn to scale). Strain identifiers are on the y-axis together with the ST131 phylogenetic tree (SNP based), coloured according to clade designations: red, clade A; orange, clade B; pink, B-C intermediate clade; green, clade C. The presence of ST131_GI-*pheV* in ST131 was identified by BLASTn analysis of ST131 genome assemblies using EC958_GI-*pheV* as a reference and a cut-off at 95% nucleotide conservation. The BLASTn data was visualized with SeqFindr. The presence of GI-*pheV* and the type of *intP4* and *attR* are indicated on the right.

Comparative genomic analysis revealed that GI-*pheV* in two completely sequenced *E*. *coli* ST131 clade C2 strains, EC958 (isolated in the UK [39]) and JJ1886 (isolated in the USA [40]), have the same gene content (Fig 3). To examine the gene content of GI-*pheV* more broadly in ST131, we used a previously published dataset comprising 102 genomes from six different geographic regions that accurately reflects the diversity and clade structure of this lineage [6,7]. A blastn search of these genomes revealed EC958_GI-*pheV* is present in 91 strains (89%), with 82 strains possessing a GI-*pheV* almost identical to EC958_GI-*pheV* (hereafter referred to as ST131_GI-*pheV*) (Fig 6). This high level of ST131_GI-*pheV* conservation occurred independent of clade phylogeny. In strains that did possess differences in ST131_GI-*pheV*, these were associated with a lack of one or more gene modules. Differences were observed in some strains from each clade. For example, in clade A strains, the *shiF-iucABCD-iutA-sat* module was absent from S31EC and S94EC, consistent with a single deletion event within their respective GI-*pheV* islands. Similarly, the *nan*, *flu*, and *yee* modules were absent from the clade C strain P50EC (Fig 6). Strain S39EC contains a remnant of ST131_GI-*pheV*, with only *intP4* and *shiA* remaining. The deletion of whole modules, but not individual genes, would be consistent with a single deletion event mediated by flanking IS sequences.

We next investigated the *intP4* gene sequences in the ST131_GI-*pheV* islands. Of 91 ST131_GI-*pheV* islands, we excluded 12 that lacked a full length *intP4* gene, presumably due to IS. Most of the remaining 79 ST131_GI-*pheV* islands harboured an identical *intP4*.*1* allele and the same 19-bp imperfect right direct repeat (*attR*) sequence as in either JJ1886_*attR* or EC958_*attR* (Fig 6). The fact that most GI-*pheV* in ST131 harbour very similar gene content, in addition to the conserved nature of their cognate *intP4* and *attR* sequences, suggests that the acquisition of ST131_GI-*pheV* into ST131 may have occurred in the common ancestor of ST131. Subsequent to this event, the deletion of individual modules or even the entire GI appears to have occurred sporadically in a strain-specific manner.

### ST131_GI-*pheV* has co-evolved with the ST131 core genome

**Fig 7:**
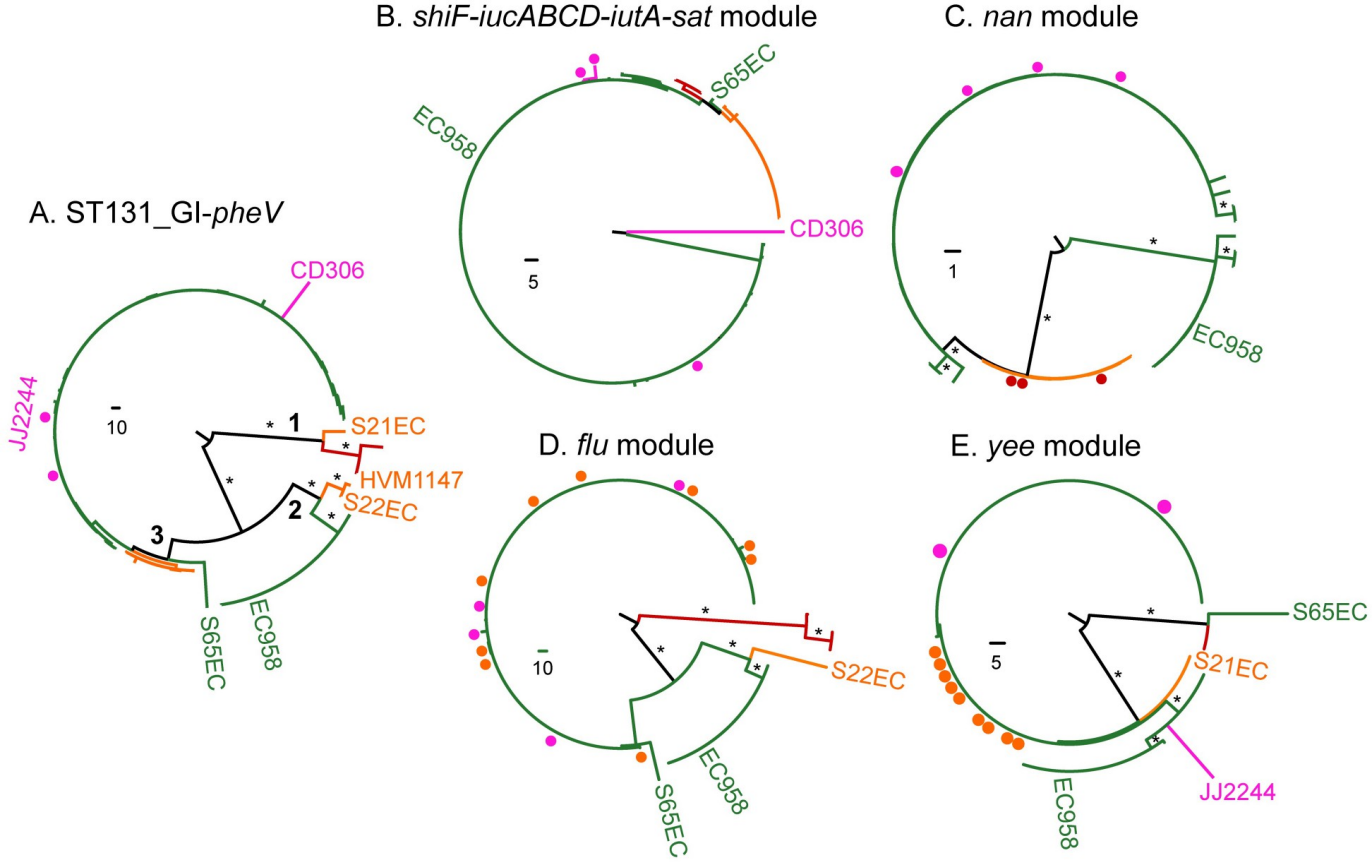
Evolutionary relationships of ST131_GI-*phe*V and their gene modules. Whole GI-*pheV* or gene modules were aligned using Mauve or ClustalO, respectively. Phylogenetic trees were constructed using RAxML, with 1,000 bootstraps. Highly supported branches are shown as asterisks for a bootstrapped value > 75%, with scales indicating the number of substitution SNPs. Branches are coloured according to the ST131 clades to which the majority of strains belong: red, clade A; orange, clade B; pink, B-C intermediate clade; green, clade C. Individual strains that cluster with strains from different clades are indicated by filled circles or strain names coloured with their corresponding clade. (A) EC958_GI-*pheV*. (B) *shiF_iucABCD_iutA_sat* module. (C) *nan* module. (D) *flu* module. (E) *yee* module.

ST131_GI-*pheV* is the predominant GI-*pheV* type in ST131, and is found across all three ST131 clades. To better understand the evolution of ST131_GI-*pheV*, we analyzed their phylogenetic relationship. Excluding the 12 ST131_GI-*pheV* that lacked a full-length *intP4* gene and 5 that had gene module deletions (S131EC, S94EC, G216, P50EC and S39EC) (Fig 6), we generated a 47,550-bp core ST131_GI-*pheV* alignment. Phylogenetic analysis of this core alignment showed that ST131_GI-*pheV* formed three well-supported groups, ST131_GI-*pheV*.1, ST131_GI-*pheV*.2 and ST131_GI-*pheV*.3 (Fig 7A). ST131_GI-*pheV*.1 are mostly found in clade A strains whereas ST131_GI-*pheV*.3 is found across clades B and C. ST131_GI-*pheV*.2 includes EC958 and 11 closely related clade C2 strains as well as a well-supported sister group of clade B strains. The fact that the ST131_GI-*pheV* phylogeny is mostly congruent with the core genome phylogeny strongly suggests this element has co-evolved with its corresponding core genome.

The phylogenetic discrepancies apparent in ST131_GI-*pheV*.2 compared to ST131 phylogeny prompted us to investigate whether there were any different evolutionary trajectories for components of GI-*pheV* across ST131. Similar to what we observed in intP4.1_GI-*pheV* from the *E*. *coli* complete genomes, there were also differences in the SNP distribution of gene modules in ST131_GI-*pheV* (Fig 7). The *shiF-iucABCD-iutA-sat* module is extremely well conserved in ST131_GI-*pheV*, with fewer than 10 SNPs over a 13,889 bp-alignment, mostly in strain CD306 (Fig 7B). Similarly, the *nan* module was conserved in ST131_GI-*pheV* except in strains carrying ST131_GI-*pheV*.2, suggesting that recombination within a hypothetical gene in the *nan* module (locus tag EC958_3299) is partly responsible for the phylogenetic placement of ST131_GI-*pheV*.2 (Fig 7C). In contrast, we observed a high SNP density in both the *flu* and *yee* modules, with 494 SNPs over 5,707-bp alignment and 142 SNPs over 2,510-bp alignment, respectively (Fig 7D and 7E). In addition, the *flu* module from a subset of 12 clade C strains containing ST131_GI-*pheV*.2 formed a distinct group, suggesting recombination has shaped its composition in these strains. Indeed, the SNP frequencies in the *flu* and *yee* modules are 8.66 x 10^−2^ and 5.66 x 10^−2^ SNPs per base pair, which are higher than the average SNP density in ST131 recombination regions (1.19 x 10^−2^) and significantly higher than that in the non-recombination regions reported previously for ST131 (1.39 x 10^−3^) [6]. Together with the *nan* module, these differences distinguish ST131_GI-*pheV*.2 from other ST131_GI-*pheV*. In all modules, increased SNP frequency was observed in several strains (e.g. CD306, S65EC, S22EC and JJ2244), providing evidence for strain-specific evolution of GI-*pheV*.

### Other GI-*pheV* types in *E*. *coli* ST131

**Fig 8:**
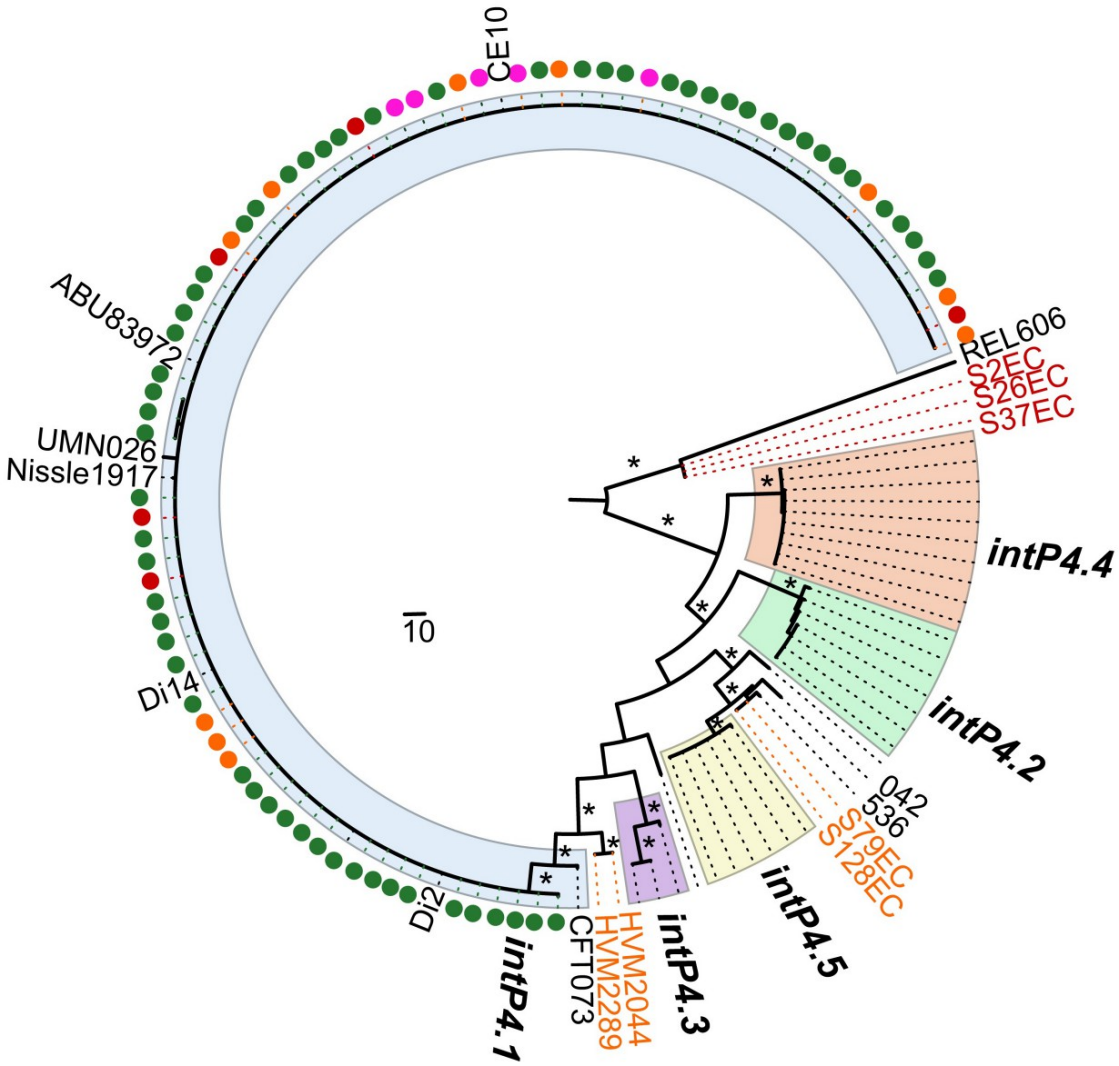
Relationship between *intP4* in ST131 and in *E*. *coli* complete genomes. A phylogenetic tree of ST131_*intP4* together with 40 *intP4* from 66 complete *E*. *coli* genomes examined in this study was constructed using RaxML, with 1,000 bootstraps. The majority of the ST131_*intP4* clustered in the *intP4*.*1* allele, together with other UPEC strains. ST131 strains are indicated by coloured dots in accordance with their clade designation: red, clade A; orange, clade B; pink, B-C intermediate clade; green, clade C. Highly supported branches are marked by an asterisk (bootstrapped value >75%).

We next investigated the presence of GI-*pheV* in 11 strains that did not contain ST131_GI-*pheV*. The presence of GI-*pheV* was manually determined based on the presence of the *intP4* gene immediately after *pheV*-tRNA. Among the 11 strains, four strains (HVM277, HVM52—clade B, H17—intermediate B-C clade and S124EC—clade C) did not harbour *intP4*, suggesting the deletion of the entire island. The *intP4* gene was found in the remaining seven strains (S2EC, S26EC and S37EC—clade A; HVM2044, HVM2289, S79EC and S128EC—clade B), indicating the presence of a GI-*pheV* with different gene content compared to ST131_GI-*pheV*. The phylogenetic relationship of *intP4* from these seven GI-*pheV* revealed they differ from *intP4* in EC958_GI-*pheV*, with the nucleotide divergence ranging from 2.6%–10.3%. Compared to *intP4* from the 66 complete *E*. *coli* genome dataset, *intP4* in S2EC, S26EC and S37EC (clade A) clustered in the same group as of REL606_*intP4*, while *intP4* in S79EC and S128EC (clade B) formed a subgroup with 536_*intP4* (Fig 8). Indeed, these two *intP4* genes differ from the *intP4* gene of the pyelonephritis strain 536 by only two SNPs. The *intP4* gene in HVM2044 and HVM2289 was closest to the *intP4*.*1* allele group, but formed a separate well-supported clade (Fig 8).

**Fig 9:**
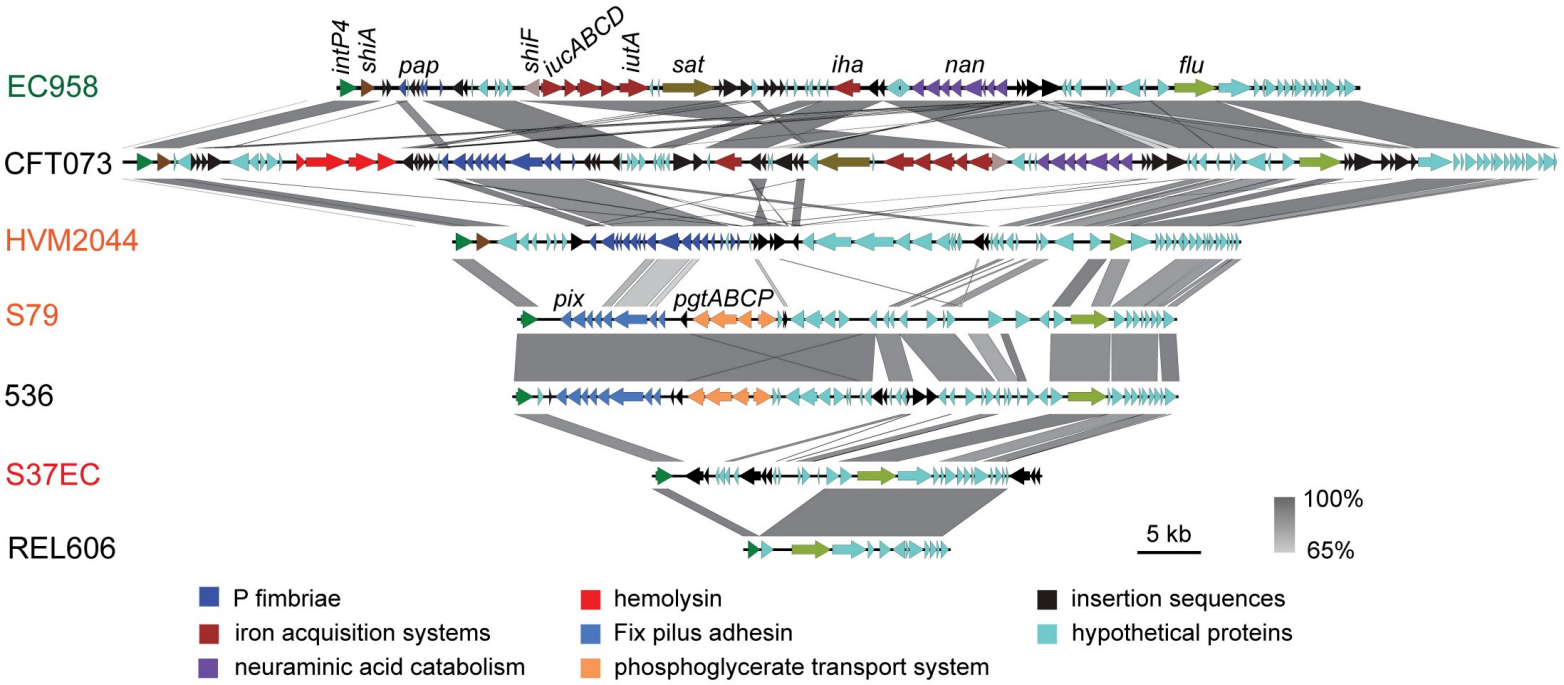
Genomic comparison among GI-*pheV* with different gene content in ST131. Various gene content of GI-*pheV* visualized based on the alignment of the entire GI using Easyfig. The black to grey gradient between the GI indicates their pairwise nucleotide conservation. Conserved genes and gene modules are coloured as indicated in the text. Strain names are coloured in accordance with their ST131 clade designation: red, clade A; orange, clade B; green, clade C.

Due to the presence of insertion sequences and repetitive regions within GI-*pheV*, we could not determine the complete gene content of GI-*pheV* in the above seven strains using *de novo* assemblies from *Illumina* sequencing alone. To overcome this problem, the complete genome of one representative strain from each different *intP4* (S37EC, S79EC and HVM2044) was determined by PacBio sequencing. Analysis of these genomes revealed the complete GI-*pheV* sequences shared little similarity with ST131_GI-*pheV*; however, their gene content was similar to other GI-*pheV* of the same *intP4* type (Fig 9). For example, S37EC_GI-*pheV* and REL606_GI-*pheV* shared 97% nucleotide sequence conservation in their respective *flu/yee* modules (Fig 9). Similarly, S79EC_GI-*pheV* and 536_GI-*pheV* both contain near identical *pix* gene clusters (encoding Pix fimbriae) and *pgtABCP* loci (encoding a phosphoglycerate transport system) downstream of their respective *intP4* allele. In addition, S79EC_GI-*pheV* had the same 23-bp imperfect right direct repeat as in 536_GI-*pheV*, which is different from that found in ST131_GI-*pheV*. Although HVM2044_*intP4* was closest to the *intP4*.*1* allele found in ST131_GI-*pheV* (Fig 8), the gene content of HVM2044_GI-*pheV* was substantially different (Fig 9). HVM2044_GI-*pheV* contained a complete *pap* gene locus (P fimbriae), a gene encoding a DNA methylase, and several regions not found in ST131_GI-*pheV*, as well as a 23-bp imperfect *attR*. Together, these data suggest that even in a single ST131 lineage, several different types of GI-*pheV* can be found. However, ST131_GI-*pheV* remains the predominant type of GI-*pheV* in ST131.

## Discussion

It is well established that GIs contribute to the evolution and pathogenesis of *E*. *coli*, but their large size and genetic diversity makes the study of their evolutionary trajectory challenging. Here we examined the evolution of GI-*pheV*, a GI integrated at *pheV*-tRNA on the *E*. *coli* chromosome.

We found that the presence of GI-*pheV* is significantly associated with pathogenic *E*. *coli* compared to non-pathogenic strains. In addition, GI-*pheV* prevalence is higher in phylogroups associated with pathogenesis compared to commensalism. In agreement with previous studies of *E*. *coli* GIs [23,41], GI-*pheV* was shown to have a mosaic structure containing gene modules flanked by insertion sequences and/or mobile elements. The gene content of GI-*pheV* is extremely diverse, with the P4-like integrase encoding gene *intP4* the only conserved gene. It has previously been shown that the GI-encoded integrases of *E*. *coli* are related to the integrase of phage P4 [34]. Our analyses identified five *intP4* alleles, the majority of which grouped in concordance with the whole genome phylogeny, suggesting co-evolution of each GI-*pheV* type with the *E*. *coli* chromosome. In addition to *intP4*, the *flu* module and partial *yee* module were found on different GI-*pheV*. These modules are also present on GIs from other species, including the pathogenicity island in *Shigella flexneri* and the cryptic prophage CP4-44 in *E*. *coli* K-12 [42,43]. Together, this suggests that the original acquisition of GI-*pheV* by *E*. *coli* could have been via bacteriophage integration at the *pheV*-tRNA locus, with subsequent events that have shaped this element including the loss of bacteriophage genes, recombination, and acquisition and deletion of gene modules.

The length and the composition of direct repeats that flank GIs are important for their mobility, and thus the integrity of these elements impacts the stability of GIs on the chromosome [44,45]. For example, a single nucleotide mismatch in the direct repeats can reduce the frequency of GI deletion by several orders of magnitude [46]. The effect on stabilization of GIs into the host chromosome is exemplified by the high-pathogenicity island (HPI) of *Yersinia enterocolitica*, which lacks intact flanking repeats and is stably maintained on the chromosome in the species [47]. We showed that GI-*pheV* that lack direct repeats are shorter than those with direct repeats likely due to ancestral deletions that removed the *attR* sites and stabilization into the host chromosome. In intP4.1_GI-*pheV*, most GIs contain a 19-bp imperfect repeat, of which *attR* has a T substitution compared to the *attL* repeat. The *attR* from EC958_GI-*pheV* also contains a second T substitution that is also found in ST131 strains that carried ST131_GI-*pheV*.2 and may be associated with increased stability compared to other intP4.1_GI-*pheV*.

Our analysis of GI-*pheV* in UPEC revealed that while its overall gene content is conserved, modules and individual genes exhibit variation suggestive of strain specific evolution. A similar phenomenon has been reported for the LEE of atypical EPEC [18]. The *flu* and *yee* genes are diverse, likely due to recombination, with only one gene showing evidence for positive selection in UMN026. In contrast, genes involved in sialic acid catabolism (*nan* module) are highly conserved (99.6% nucleotide conservation). Sialic acid is abundant in human mucus and can be utilized by *E*. *coli* as a sole source of carbon and energy. Sialic acids also contribute to the regulation of type 1 fimbriae, providing a mechanism to balance pathogen-host interactions [48]. Together, the conserved *nan* modules among GI-*pheV* in UPEC may enhance its capacity to colonise host epithelial surfaces, such as in the intestinal and urinary tract. Similarly, the *iucABCD* genes encoding the aerobactin system involved in iron acquisition are largely conserved and may contribute to UPEC virulence. However, a divergent region of sequence overlaps both the ferric aerobactin receptor gene *iutA* and the upstream *iucD* gene in CFT073 and UMN026 compared to other strains that contain the intP4.1_GI-*pheV*. The phylogenetic distance between CFT073 and UMN026 and the high nucleotide identity shared by their respective *iucD/iutA* regions (and, indeed, the rest of their respective GI-*pheV* sequences) are consistent with recombination and subsequent lateral gene transfer of GI-*pheV*. Although we did not find any evidence for positive selection in *iutA*, our data suggest that it may have evolved independently within this module in some UPEC strains. Polymorphisms in *iutA* have also been observed in other *E*. *coli* pathotypes, *Shigella* spp., *Klebsiella pneumoniae* and *Vibrio hollisae* [49–51].

ST131 was initially identified as a clone associated with the production of CTX-M-15 [52–54]. Since then, ST131 has become recognised globally as one of the most successful ExPEC lineages [6,7,10,55,56]. Here we investigated GI-*pheV* in a previously validated ST131 genome dataset, and revealed dynamic changes in this element over a short evolutionary timespan. GI-*pheV* was present in 96% of the ST131 isolates investigated, most of which contained a GI-*pheV* similar to that found in the reference ST131 strain EC958. These ST131_GI-*pheV* islands possess a near-identical *intP4*.*1* allele (nucleotide divergence of 1–2 nonsynonymous SNPs) and highly conserved 19-bp direct repeats. Furthermore, the ST131_*GI-pheV* islands were found across all ST131 clades, including a clade B-C intermediate strain JJ1897.

Different levels of diversity were observed among the GI-*pheV* gene modules in the ST131 strains examined. The *iucABCD-iutA* and *nan* module displayed very high conservation, while the *flu* and *yee* modules were the most variable. Although the ST131_GI-*pheV* was the most common, an additional three different types of GI-*pheV* were also identified in ST131. These GI-*pheV* possess distinct types of *intP4* and direct repeats, consistent with their independent acquisition. The strains that harbour these alternate GI-*pheV* generally clustered together with other strains that lack a GI-*pheV* altogether. For example, HVM2044 and HVM2289, which have a GI-*pheV* similar to 536_GI-*pheV*, clustered with HVM277 and HVM52, which do not have GI-*pheV*. Based on these data, we propose the following evolutionary trajectory of GI-*pheV* in ST131: (i) the acquisition of ST131_GI-*pheV* in the common ancestor of ST131; (ii) co-evolution of ST131_GI-*pheV* in the three major ST131 clades, including recombination within gene modules; (iii) sporadic loss of gene modules or the entire GI; and (iv) the acquisition of other GI-*pheV* types by some strains that had previously lost the ST131_GI-*pheV*.

In summary, this work presents a comprehensive evolutionary study of the GI-*pheV* element. While the GI-*pheV* gene content exhibits extensive diversity, we observed that this follows a pathotype-specific association. In UPEC, GI-*pheV* contains a number of virulence and fitness modules linked to colonization of the urinary tract. The evolution of GI-*pheV* in ST131 is more complex, with our analysis revealing it was acquired early in the evolution of ST131, with loss or gain of genes and modules via recombination shaping its composition.

## Materials and methods

### *E*. *coli* strains and genomic data

The 2,382 *E*. *coli* complete genomes were retrieved from NCBI Reference Sequence Database on 23/12/2022 (S1 Table). The reference set of 66 completely sequenced *E*. *coli* genomes comprising 23 non-pathogenic and 43 pathogenic strains (28 intestinal and 15 extra-intestinal) was downloaded from Genbank (S2 Table). *In silico* multi-locus sequence typing (MLST) was performed according to the seven-gene scheme [57]. Read data and *de novo* assemblies of 95 *E*. *coli* ST131 were obtained from previous studies [6,7]. The genome sequences of an additional seven strains [58] that represented an intermediate link between ST131 strains from clades B and C [7] were also analysed. *De novo* assemblies were ordered against the genome of the reference ST131 strain EC958 [59] using Mauve [60].

Three ST131 strains (HVM2044, S79EC and S37) that harboured GI-*pheV* and exhibited extensive diversity compared to the EC958 ST131_GI-*pheV* were sequenced on a PacBio RS II sequencing instrument using 3 SMRT cells, using a 10-kb insert library and the P6-C4 sequencing chemistry. *De novo* genome assembly was performed using PacBio’s SMRT Portal (v2) and the hierarchical genome assembly process (HGAP v2.0) with default settings and a seed read cut-off length of 5 kb. The complete genome sequences were annotated using Prokka [61] and insertion sequence (IS) annotation was done with ISFinder (https://www-is.biotoul.fr/). Annotation of CDS and IS were then curated manually, with a focus on GI-*pheV*. PacBio reads were deposited in the SRA with the following accession numbers: SRR10761087, SRR10761088 and SRR10761089.

### GI-*pheV* identification and comparative genomic analysis

The prevalence of GI-*pheV* in the 2,382 *E*. *coli* complete genomes was evaluated based on the presence of the gene located upstream of *pheV*-tRNA, *pheV*-tRNA and the integrase *intP4* gene. The 41 GI-*pheV* from the reference set of 66 *E*. *coli* complete genomes were manually identified based on the presence of an integrase encoding gene at the 3’ end of the *pheV*-tRNA. The 3’ end of GI-*pheV* was determined based on the presence of a direct repeat sequence (*attR*) that represents the remnant of *pheV*-tRNA. Where a direct repeat was not identified, an approximation of the 3’ end of GI-*pheV* was determined based on comparison to the genome of the *E*. *coli* K-12 strain MG1655, and was determined as the last nucleotide before the conserved region found in the same location in MG1655 or at the breakpoint before the *kps* locus. The gene content of each GI-*pheV* was compared using Mauve, ACT and EasyFig [60,62,63].

The prevalence of ST131_GI-*pheV* in the sequenced ST131 strain set was examined using BLASTn [64] and visualized with SeqFindr (https://github.com/mscook/seqfindr). The presence/absence of GI-*pheV* was presented to scale by performing analyses over a 200 bp sliding window, applying a nucleotide identity cut-off at 0.95. The locations of GI-*pheV* in the *E*. *coli* ST131 draft genomes were identified manually. Briefly, the contigs from *de novo* assemblies of 102 *E*. *coli* ST131 strains were ordered against the complete EC958 genome. GI-*pheV* was then annotated based on the presence of the *intP4* gene located downstream of *pheV*-tRNA. The end of the GI-*pheV* was defined based on pairwise comparison to EC958_GI-*pheV*. The direct repeat *attR* was identified by manually searching for *attR* sequences listed in Table 1.

### Sequence alignment and phylogenetic analysis

GI-*pheV* nucleotide sequences from complete genomes were aligned using Mauve [60]. Regions larger than 100 bp were extracted from the alignment and concatenated using the stripSubsetLCBs script supplied by Mauve to generate the core GI-*pheV* alignment. Individual genes and regions of interest were aligned using ClustalO and visualized using SeaView [65,66]. The ratio of non-synonymous to synonymous substitutions (dN/dS ratio) was calculated with SNAP [67,68] using codon-aligned nucleotide sequences of individual genes generated by SeaView program [66]. The dN/dS ratio is used to indicate if a gene is conserved or may have undergone adaptive evolution, with dN/dS > 1 indicating positive (adaptive or diversifying) selection, dN/dS = 1 indicating neutral evolution, and dN/dS < 1 indicating negative (purifying) selection. Phylogenetic trees of the core GI-*pheV* alignments and other alignments were constructed with RaxML v.7.2.8 using substitution-only single nucleotide polymorphisms (SNPs) with the general time reversible (GTR) GAMMA model of among site rate variation (ASRV) [69]. The robustness of the trees was tested using 1,000 bootstraps. Trees were visualized and edited using FigTree v1.3.1 (http://tree.bio.ed.ac.uk/software/figtree/) as previously described [70–72].

## Supporting information

S1 Fig Prevalence of GI-*pheV* in STs represented in the 2,382 *E*. *coli* complete genomes from RefSeq Database. Bars are coloured according to the corresponding phylogroup. The total number of *E*. *coli* genomes in each ST is shown in brackets.
(TIF)

S2 Fig Pangenome of GI-*pheV*. Gene presence and absence of GI-*pheV* from 40 *E*. *coli* complete genomes, together with their GI-*pheV-intP4* phylogeny.
(TIF)

S3 Fig Sequence conservation of *intP4* in GI-*pheV* from the 1,242 GI-*pheV*-positive *E*. *coli* complete genomes. Shown is the percentage nucleotide identity of *intP4* in GI-*pheV* from different phylogroups (A) and STs (B).
(TIF)

S4 Fig Comparison of gene content in representative GI-*pheV* from different *intP4* alleles. Nucleotide comparison of a representative GI-*pheV* from each *intP4* allele revealed extensive diverse, with *intP4* as the only conserved core gene. The outer ring was coloured according to *intP4* alleles as in Fig 1 (blue, *intP4*.*1*; green, *intP4*.*2*; violet, *intP4*.*3*, light orange, *intP4*.*4* and yellow, *intP4*.*5*). Inner rings are regions on each GI-*pheV* coloured according to the following scheme: blue, virulence/fitness factors; orange, insertion sequences and mobile elements; light grey, hypothetical proteins. Nucleotide sequence conservation is shown as ribbons coloured green (indicating 90–100% conservation) and red (indicating 80–89% conservation).
(TIF)

S5 Fig Gene contents of GI-*pheV* with different *intP4* alleles from *E*. *coli* complete genomes. Pairwise comparison of GI-*pheV* having the same *intP4* allele, visualized by Easyfig, with black to grey gradient shows the percentage of nucleotide similarity. Strains with bolded name were compared in S4 Fig.
(TIF)

S1 Table Details of the 2,382 *E*. *coli* complete genomes used in this study. (XLSX)

S2 Table Details of the reference set of 66 completely sequenced *E*. *coli* genomes used in this study. (XLSX)

S3 Table SNP distribution in gene modules and individual genes of intP4.1_GI-*pheV*. (XLSX)
