# Supplementary figures and images for: Evolution of the pheV-tRNA integrated genomic island in Escherichia coli
Source: PLoS Genet. 2024 Oct 24;20(10):e1011459. doi: 10.1371/journal.pgen.1011459 (PMC11537424; doi:10.1371/journal.pgen.1011459)

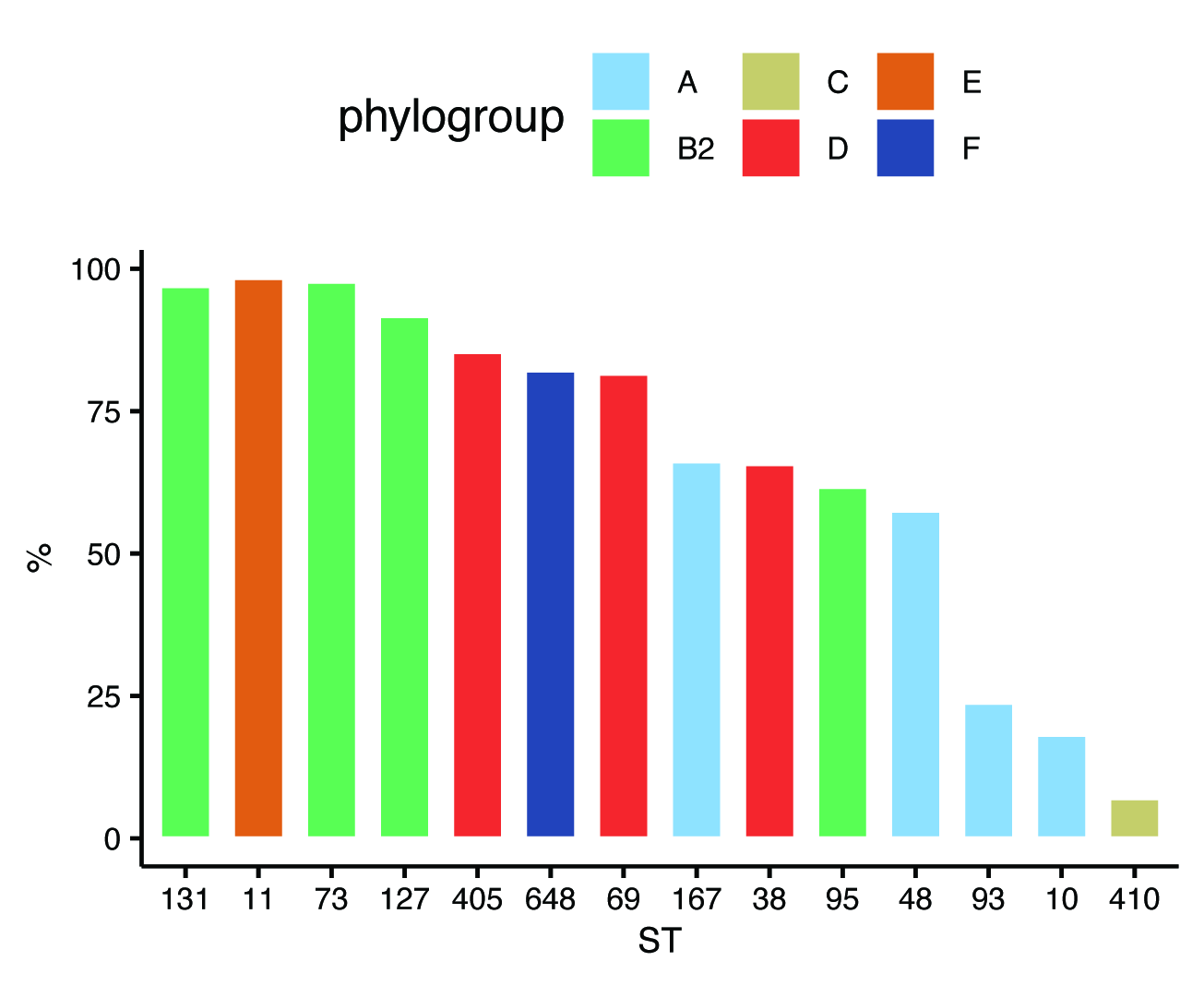

Supplement: S1 Fig — Bars are coloured according to the corresponding phylogroup. The total number of E. coli genomes in each ST is shown in brackets. (TIF) [file pgen.1011459.s001.tif]

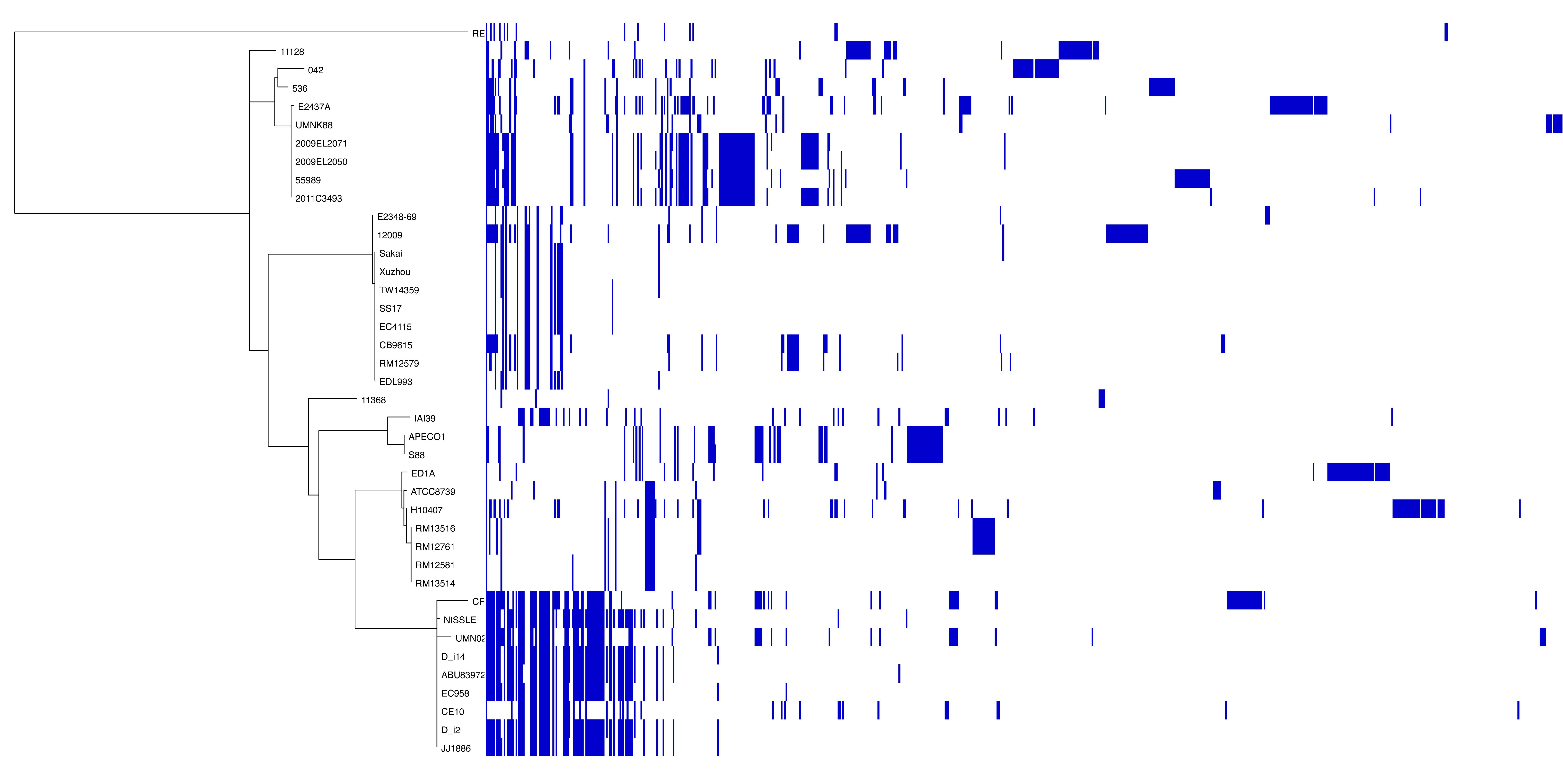

Supplement: S2 Fig — Gene presence and absence of GI-pheV from 40 E. coli complete genomes, together with their GI-pheV-intP4 phylogeny. (TIF) [file pgen.1011459.s002.tif]

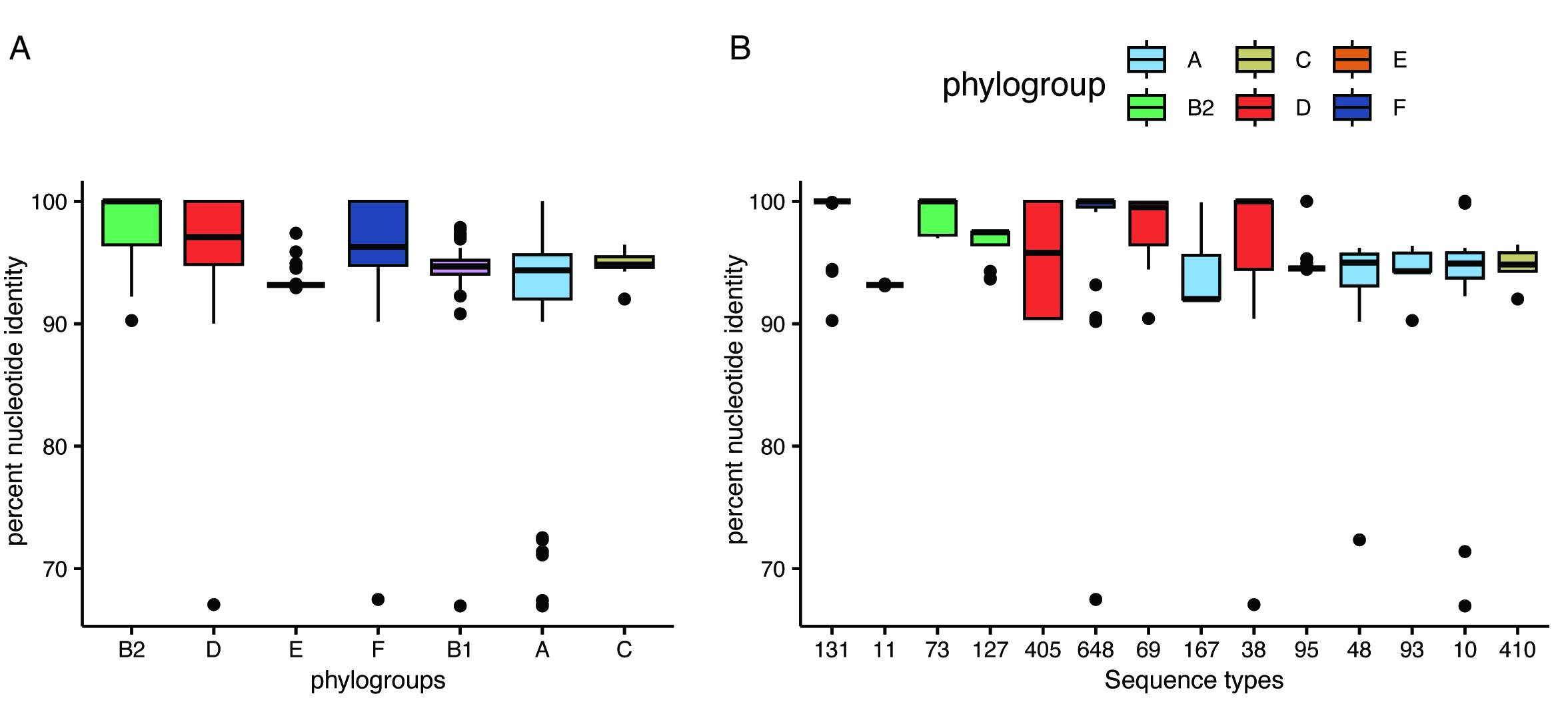

Supplement: S3 Fig — Shown is the percentage nucleotide identity of intP4 in GI-pheV from different phylogroups (A) and STs (B). (TIF) [file pgen.1011459.s003.tif]

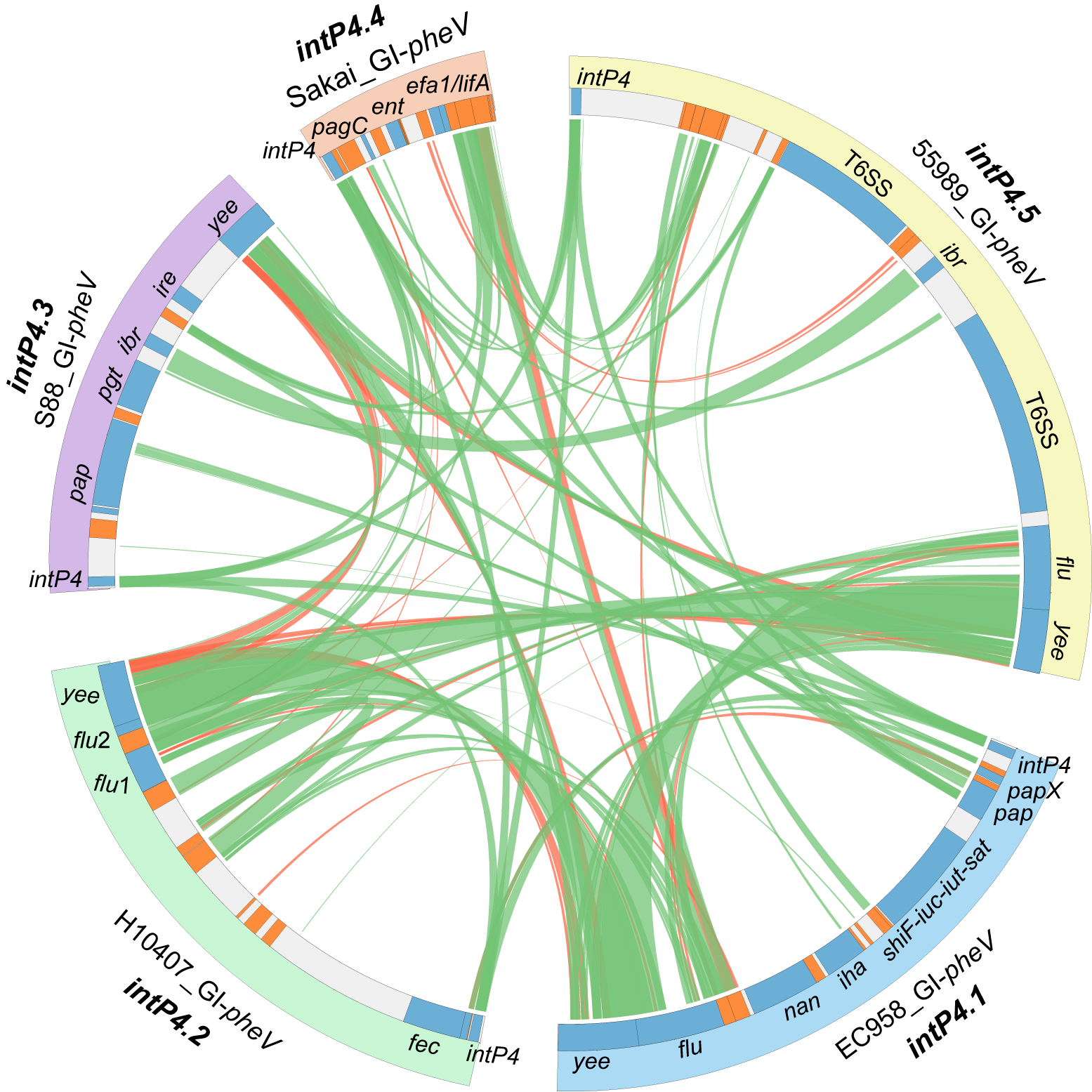

Supplement: S4 Fig — Nucleotide comparison of a representative GI-pheV from each intP4 allele revealed extensive diverse, with intP4 as the only conserved core gene. The outer ring was coloured according to intP4 alleles as in Fig 1 (blue, intP4.1; green, intP4.2; violet, intP4.3, light orange, intP4.4 and yellow, intP4.5). Inner rings are regions on each GI-pheV coloured according to the following scheme: blue, virulence/fitness factors; orange, insertion sequences and mobile elements; light grey, hypothetical proteins. Nucleotide sequence conservation is shown as ribbons coloured green (indicating 90–100% conservation) and red (indicating 80–89% conservation). (TIF) [file pgen.1011459.s004.tif]

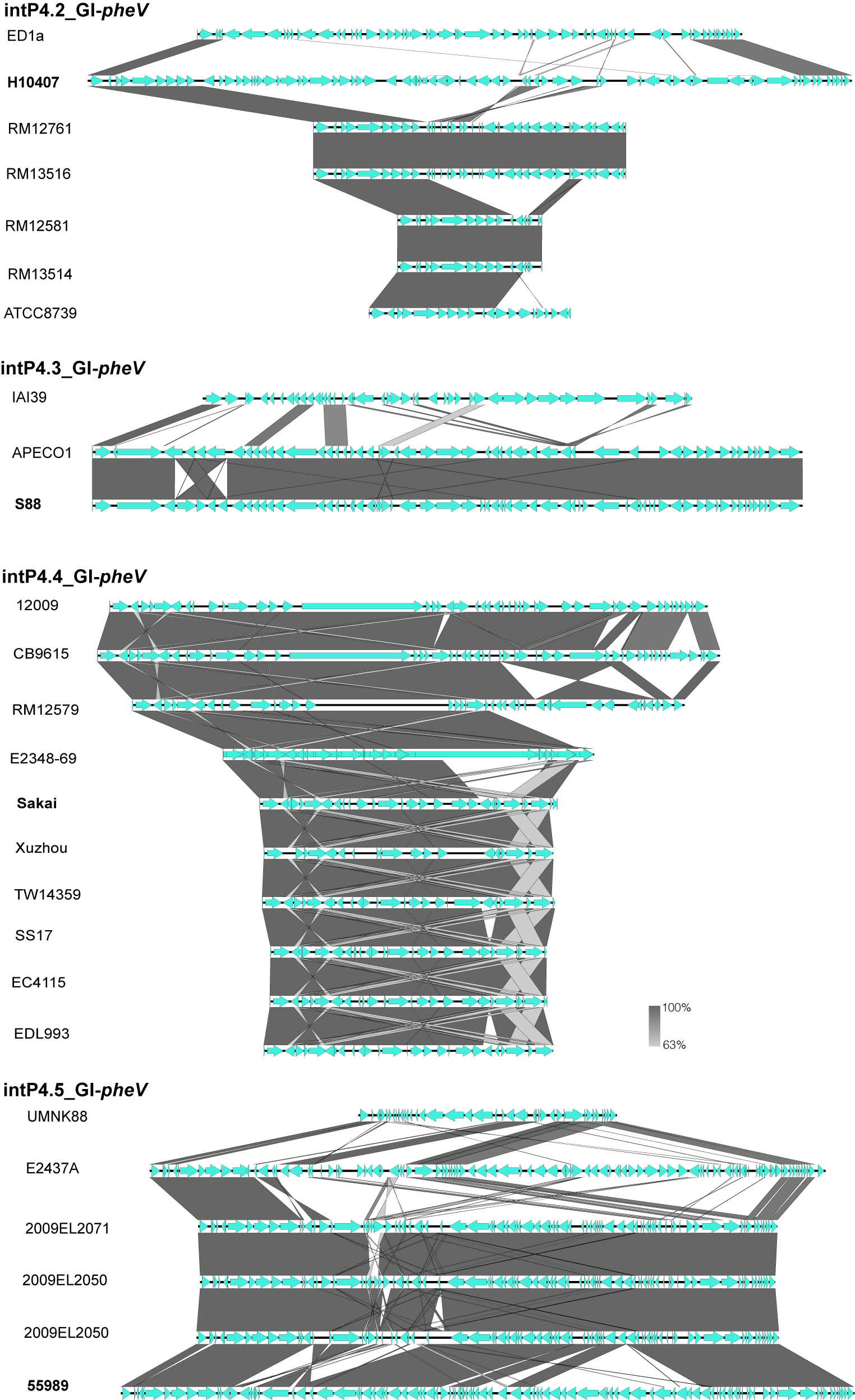

Supplement: S5 Fig — Pairwise comparison of GI-pheV having the same intP4 allele, visualized by Easyfig, with black to grey gradient shows the percentage of nucleotide similarity. Strains with bolded name were compared in S4 Fig. (TIF) [file pgen.1011459.s005.tif]
